# Effect of recombinant and native buffalo OVGP1 on sperm functions and in vitro embryo development: a comparative study

**DOI:** 10.1186/s40104-017-0201-5

**Published:** 2017-09-01

**Authors:** Suman Choudhary, A. Kumaresan, Manish Kumar, Shivani Chhillar, Hrudananda Malik, Sudarshan Kumar, Jai K. Kaushik, Tirtha K. Datta, Ashok K. Mohanty

**Affiliations:** 1 Animal Biotechnology Centre, National Dairy Research Institute, Karnal, Haryana 132001 India; 2 Livestock and Production Management Section, National Dairy Research Institute, Karnal, Haryana 132001 India

**Keywords:** Blastocyst, Capacitation, Fertilization, Glycoprotein, In vitro fertilization, Oestrus, Semen, Sperm

## Abstract

**Background:**

An oviduct- specific glycoprotein, OVGP1, is synthesized and secreted by non-ciliated epithelial cells of the mammalian oviduct which provides an essential milieu for reproductive functions. The present study reports the effects of recombinant buffalo OVGP1 that lacks post-translational modifications, and native Buffalo OVGP1 isolated from oviductal tissue, on frozen- thawed sperm functions and in vitro embryo development.

**Results:**

The proportion of viable sperms was greater (*P* < 0.05) in the recombinant OVGP1-treated group compared to the native OVGP1-treated group at 2 h, 3 h, and 4 h of incubation. The proportion of motile sperms at 3 h and 4 h of incubation; and membrane- intact sperms at 4 h was greater (*P* < 0.05) in the native OVGP1-treated group compared to the control and recombinant OVGP1-treated groups. The proportion of capacitated and acrosome- reacted sperms was greater (*P* < 0.05) in the native OVGP1-treated group compared to the recombinant OVGP1 group at 4 h. The rates of cleavage of embryos and their development to the blastocyst stage were greater (*P* < 0.05) in the presence of either native or recombinant OVGP1 in comparison to control at 10 μg/mL concentration as compared to 5 or 20 μg/mL.

**Conclusions:**

The study suggests that both native and recombinant OVGP1 impart a positive effect on various sperm features and in vitro embryo development. However, native OVGP1 was found to have a more pronounced effect in comparison to recombinant non-glycosylated OVGP1 on various sperm functions except viability. Hence, our current findings infer that glycosylation of OVGP1 might be essential in sustaining the sperm functions but not the in vitro embryo development.

**Electronic supplementary material:**

The online version of this article (doi:10.1186/s40104-017-0201-5) contains supplementary material, which is available to authorized users.

## Background

The mammalian oviduct is an active secretory organ, which undergoes significant endocrine-induced morphological, biochemical and physiological changes to establish an essential microenvironment within the oviduct for final maturation of gametes, capacitation of sperm, transport of gametes and embryos, fertilization and early cleavage-stage embryonic development [1]. Specific macromolecular components are secreted within the oviduct and interact with gametes and embryos [2]. OVGP1 i.e. Oviduct- specific glycoprotein was reported as the major secretory glycoprotein which is synthesized and secreted exclusively by the oviduct [1]. It was identified in many species like sheep [3], hamster [4], human [5], mouse [6], cattle [7], pig [8], goat [9], baboon [10], rhesus monkey [11]. OVGP1 is synthesized de novo under oestrogen control by non-ciliated epithelial cells of the oviduct and localized in the apical secretory granules followed by its release into the lumen [12]. It has been found to interact with the zona pellucida (ZP) and also observed in the perivitelline space of oocytes and embryos during fertilization in various species i.e. porcine, baboon, sheep, golden hamster [13–16]. Incubation of human and baboon ovarian oocytes in culture medium supplemented with OGP (oviduct- specific glycoprotein) resulted in binding of OGP to ZP and subsequently enhanced human sperm binding [17]. OVGP1 enhances the rate of in vitro fertilization and embryonic development in goats [18]. When gametes were pretreated with OVGP1, the fertilization rate in cattle increased significantly [19]. The addition of oviductal fluid in the incubation medium was shown to induce sperm capacitation in buffalo [20], boar [21] and cattle [22]. A recent study reported that oviductal fluid modulates tyrosine phosphorylation of sperm proteins [23]. The existing information indicates that OVGP1 purified from the oviduct has positive effects on sperm capacitation, sperm-ovum binding, ovum penetration and embryonic development. Bovine oviduct specific sialo-glycoprotein exists in different glycosylated forms due to post- translational modifications which may provide specific biological properties to the protein [24]. These modifications may influence protein–protein or protein–cell interactions with sperm membranes, zonae pellucidae, the vitelline membrane of oocytes, or the blastomere membrane of early embryos.

A recent study has demonstrated that OVGP1 binds to ZP through the N- terminal region of OVGP1 in porcine and rabbit, and is endocytosed by the oocyte after penetrating the ZP through its C- terminal region, leading to an increase in the in vitro fertilization (IVF) efficiency [25]. Reproductive efficiency in buffaloes is hampered by various factors such as natural late maturity, poor expression of estrus in summer, prolonged intervals from calving to estrus, and distinct seasonal patterns of reproduction hamper the reproductive efficiency in buffalo [26].An efficient reproductive performance in buffaloes is required for harvesting their maximum reproductive and productive potential. The technology of in vitro embryo production was reported to have limited success in buffalo primarily because of a low rate of blastocyst formation in comparison to other species like cattle and goat [27–29]. The information on the effect of native OVGP1 on sperm functions and embryonic development is available to some extent. However, the effect of recombinant buffalo OVGP1 without post- translational modifications in comparison to native OVGP1 is yet to be elucidated. Furthermore, elucidation of the biological functions and mechanism regulating the functions of OVGP1 is hampered by limited quantities of OVGP1 that can be purified from oviductal fluid and/or oviductal cells. A comparative study of effects of native and recombinant OVGP1 on sperm functions and in vitro embryo development needs to be performed to gain insights into the role of carbohydrate moieties. In the present investigation, we report the purification of recombinant buffalo OVGP1 expressed in *E. coli* and native buffalo OVGP1 isolated from oviducts along with their effects on sperm functions and in vitro embryo development. These findings advance our understanding on the importance of glycans attached to native OVGP1 in comparison to recombinant OVGP1 in influencing its biological functions.

## Methods

### Chemicals and reagents

The molecular biology grade chemicals and reagents used were from Sigma Aldrich (St. Louis, MO, USA) unless otherwise mentioned. Disposable plastic ware used was from Falcon NJ, the USA, and Nunc, Denmark. Fetal bovine serum used was from Hyclone, Canada.

### Purification of recombinant OVGP1

Recombinant buffalo OVGP1 was expressed in *E. coli* and purified to homogeneity as described in our previous paper [30] with some modifications. Briefly, buffalo *OVGP1* gene was cloned into TA cloning vector and transformed into TOP10 cells. Further, subcloning was done into the pET28b(+) expression vector using Nco1 and Sal1 restriction sites and transformed into *E. coli* BL 21 (DE3) C+ cells as an expression host. The positive clones were grown till an optical density of 0.6 was reached at 600 nm and induced with 0.4 mmol/L IPTG (Isopropyl β-D-1-thiogalactopyranoside) at 37 °C with shaking at 200 r/min. Soluble protein was extracted by using Qproteome Bacterial Protein prep kit (Qiagen, USA) according to manufacturer’s instructions. The soluble and insoluble fractions were run on 12% SDS- PAGE gel. For purification of soluble recombinant OVGP1, BL 21 (DE3) C+ cells harboring OVGP1 in pET28b(+) expression construct was cultured for about 3 h in LB broth at 37 °C with 50 μg/mL kanamycin. Cells were harvested by centrifugation (4,660 ×g for 15 min), and the cell pellet was suspended in phosphate buffer saline. Cell lysis was carried out by adding lysozyme from chicken egg white (10 μg/mL) followed by incubation at room temperature for 1 h. Further lysis was done by sonication on ice at pulse intervals of 3 s on and 8 s off for 15 min. The protease inhibitor i.e. Phenyl methyl sulfonyl fluoride (PMSF) was also added before cell lysis to prevent any degradation of the protein by proteases. After lysis, Triton-X 100 being a strong detergent was added for 0.5 h to remove any membrane bound protein. Soluble and insoluble fractions were separated by centrifugation (13,680 ×g for 30 min) at 4 °C. The supernatant was loaded onto 5 mL cobalt HisTALON™ superflow cartridge (Clontech, USA) for purification of His-tagged OVGP1. After equilibration of the column, the protein was allowed to bind at a flow rate of 0.5 mL/min with a binding buffer consisting of 50 mmol/L sodium phosphate buffer, 0.3 mol/L NaCl, 10 mmol/L imidazole pH 8.0 followed by washing with the same buffer. The bound protein was eluted with elution buffer consisting of 300 mmol/L imidazole in the same wash buffer used at pH 8.0 and was subjected to SDS- PAGE analysis to check the purity. For further purification, the dialyzed eluted protein was loaded onto the 1 mL HiTrap Q HP column (GE Healthcare) pre- equilibrated with 50 mmol/L Tris buffer containing 50 mmol/L NaCl, pH 7.5. After washing with the same buffer, the adsorbed proteins were eluted with the elution buffer 50 mmol/L Tris containing 1 mol/L NaCl, pH 7.5 by applying a step wise gradient i.e. 0–30% NaCl (30 mL) and then 30–50% NaCl (50 mL). Finally, to remove the additional non- specific proteins, the eluate from anion exchange column was dialyzed and loaded onto the 20 mL Superdex 75 column (GE Healthcare) in 50 mmol/L Tris buffer containing 0.15 mol/L NaCl, pH 7.5 at a flow rate of 0.3 mL/min. The purified fractions were pooled, concentrated and desalted using stirred ultrafiltration cell (Amicon, USA) and subjected to SDS-PAGE. Gels were analyzed by Coomassie Brilliant Blue staining or transferred to nitrocellulose membrane for Western blot analysis. For Western blot, the membrane was incubated with Anti - 6 × His Epitope Tag mouse antibody (Pierce, Thermo Scientific) at 1:1,000 dilutions. The secondary antibody was horseradish peroxidase-conjugated goat anti-mouse IgG (Bangalore Genei, India) used at 1:1,000 dilutions. Immunoreactivity was detected by using the DAB (3, 3′- Diaminobenzidine) system (Bangalore Genei, India).

### Purification of native OVGP1

Native buffalo OVGP1 was purified according to the previously described protocol [31] with minor modifications. Buffalo oviductal tissue was collected from a local slaughter house in saline, thoroughly minced and then suspended in Tris buffered saline (TBS) (pH 7.4) containing 1 mmol/L PMSF followed by 3–4 cycles of freeze and thaw for lysis. The oviductal extract was centrifuged (1,500 ×g for 30 min) at 4 °C. The resulting supernatant was centrifuged again (20,000 ×g for 1 h) and was loaded onto an 8 mm × 45 mm Wheat Germ Lectin-CL Agarose affinity column equilibrated with TBS, pH 7.4 containing 1 mmol/L PMSF. The column was washed with the same buffer till the OD decreased to 0.05 at 280 nm. The bound protein was eluted with 100 mmol/L N-acetyl-D-glucosamine in TBS and dialyzed overnight at 4 °C against buffer A (50 mmol/L Tris-HCl, 100 mmol/L NaCl, pH 7.4). After dialysis, the sample was concentrated by stirred ultrafiltration cell (Amicon, USA) using 10 kDa cut off membrane. The concentrated protein was loaded onto a Mono-Q anion exchange column (GE Healthcare) pre-equilibrated in buffer A and was eluted with a linear gradient of 100 to 500 mmol/L NaCl in 50 mmol/L Tris- HCl, pH 7.4 at a flow rate of 0.5 mL/min. The fractions representing distinct peaks were subjected to SDS-PAGE to check the level of purity of native OVGP1 and were finally confirmed by Western blot. For Western blot, the membrane was incubated with goat polyclonal antibody named as oviductin (N-20) (Santa Cruz Biotechnology, INC.) at 1: 500 dilutions. Oviductin (N-20) is an affinity purified goat polyclonal antibody raised against a peptide mapping at the N-terminus of OVGP1 (also designated Mucin 9) of human origin. The secondary antibody was horseradish peroxidase-conjugated anti-goat IgG (Bangalore Genei) at 1:1,000 dilutions. Immunoreactivity was detected by using the DAB system (Bangalore Genei, India).

### Mass spectrometry (LC- MS/MS) analysis of purified recombinant and native OVGP1

Mass Spectrometry of purified recombinant and native proteins was carried out to reveal their true identity. The bands corresponding to each protein were cut out and processed by in- gel tryptic digestion. The digested peptides were reconstituted in 0.1% formic acid in LC-MS grade water and subjected to nano-LC (Nano Advance, Bruker, Germany) followed by identification by captive spray-Maxis-HD qTOF (Bruker, Germany) mass spectrometer (MS) with high mass accuracy and sensitivity. The peptides were enriched in nano trap column (Thermo Scientific) and eluted on to analytical column (Agilent) using a linear gradient of 5–45% acetonitrile at 400 nL/min over 65 min. Positive ions were generated by electro spray and the qTOF operated in data dependent acquisition mode. Data analysis was performed using MS program Mascot (2.4.1 Matrix Science, UK).

### Effect of recombinant and native OVGP1 on sperm functions

Semen processing and incubation with recombinant and native buffalo OVGP1.

The previously frozen semen from three different freezing operations (three different ejaculates) each from four Murrah buffalo bulls were used for the experiment. After thawing at 37 °C for 30 s, semen samples from each bull were pooled separately. The pooled semen was washed twice by centrifugation (300 ×g for 10 min) in phosphate buffer saline (pH 7.3). The sperm pellet was suspended in non- capacitating medium (NCM) containing 2.7 mmol/L KCl, 1.5 mmol/L KH_2_PO_4_, 8.1 mmol/L Na_2_HPO_4_, 137 mmol/L NaCl, 5.55 mmol/L glucose and 1 mmol/L sodium pyruvate (pH 7.4). The sperm suspensions were divided into three different aliquots each containing about 100 million sperm cells. The first group i.e. control group contained NCM without native and recombinant OVGP1, second, NCM with native OVGP1 (500 μg/mL), and third, NCM with recombinant OVGP1 (500 μg/mL). Control and treated groups were incubated for 4 h at 38 °C under 5% CO_2_. The aliquots were removed from incubation at hourly intervals and subjected to different in vitro sperm- function assays to observe the effect of recombinant and native OVGP1.

#### Assessment of sperm motility

Thawed semen samples were processed and treated with native and recombinant OVGP1 as mentioned above. The different aliquots for control, native and recombinant OVGP1-treated groups were removed from the incubator at hourly intervals. The sperm sample (5 μL) from each group was placed on a pre- warmed (38 °C) glass slide and covered with a cover slip. The sperm motility was assessed at 400× magnification using a phase contrast microscope (Nikon Eclipse E600, Tokyo, Japan) equipped with a stage warmer (38 °C). All motility estimations were carried out by a single, well-experienced person, without the knowledge of treatments (blind evaluation).

#### Sperm viability assessment

SYBR- 14/ PI staining was performed according to the previously described protocol [32]. Briefly, SYBR-14 (Invitrogen, USA) was diluted (1: 10) with anhydrous di- methyl sulphoxide (DMSO) to prepare the working solution. The three different aliquots of control, native and recombinant OVGP1-treated groups were taken at hourly intervals of 4 h incubation period, mixed with the working solution (5 μL) of SYBR 14 dye and incubated for 15 min at 37 °C. Subsequently, 2 μL of propidium iodide (PI) stock solution (Invitrogen, USA) was mixed and incubated at the same temperature for 5 min. The mixture was then smeared as a thin layer on a glass slide and covered with cover slip after adding the DABCO (1, 4-diazabicyclo [2.2.2] octane), an antifading agent. The sperms were assessed using a fluorescent microscope (Nikon ECLIPSE Ti-s, Japan) to determine if their membranes were intact (green) and not intact (red).

#### Sperm membrane integrity assessment

The carboxyfluorescein-diacetate-propidium iodide (CFDA-PI) staining was performed according to the previously described method in buffaloes [33] with minor modifications. The three aliquots with control, native and recombinant OVGP1-treated groups were removed from the incubator at each hourly interval of 4 h incubation period. From each group, about 10 million sperms were taken in 1.5 μL amber color eppendorf tube, 5 μL of CFDA (0.5 mg/mL) was added under the dark condition and incubated at 37 °C for 15 min before adding 2 μL PI (0.3 mg/mL). After incubation for 2 min, NCM (100 μL) was added and centrifuged (800×*g* for 3 min). After discarding the supernatant, a thin smear was made out of the pellet and antifading agent DABCO dissolved in glycerol: phosphate buffered saline (9:1) was added to the smear before applying a cover slip. The assessment was done for three semen populations i.e. membrane intact (green-stained), dead (red- stained) and moribund (doubly stained).

#### Assessment of sperm capacitation status

Chlortetracycline (CTC) staining was carried out according to the previously described protocol [34]. Chlortetracycline solution (750 mmol/L CTC, 5 mmol/L cysteine in 130 mmol/L NaCl and 20 mmol/L Tris-HCl, pH 7.4) was freshly prepared, and pH was adjusted to 7.8. The control and treated groups were taken at each hourly interval during 4 h incubation period, mixed with an equal volume of CTC solution and after a few seconds, 1 mL of 4% paraformaldehyde was added. A thin smear was made on a glass slide, and a drop of DABCO was added before applying the cover slip to retard the fading of the CTC fluorescence. Chlortetracycline fluorescence was observed using an epifluorescent microscope (Olympus, USA). The assessed sperms were classified into either uniform bright fluorescence over the whole head (uncapacitated sperm, pattern F), a fluorescence-free band in the post-acrosomal region (capacitated sperm, pattern B) and dull fluorescence over the entire head (acrosome- reacted sperm, pattern AR).

### Effect of recombinant and native buffalo OVGP1 on in vitro embryo development

#### Collection of buffalo ovaries

Buffalo ovaries were collected from a local slaughter house and transported to the laboratory in a thermal flask containing normal saline (0.9% NaCl) fortified with penicillin (400 IU/mL) and streptomycin (50 μg/mL) and maintained at 37 °C temperature.

#### Aspiration and grading of oocytes

Ovaries were washed several times in normal saline and cumulus oocyte complexes (COCs) were aspirated from visible ovarian surface follicles with the help of 18 gauge needle attached to 10 mL syringe (BD discard it) in HEPES-buffered hamster embryo culture (HH) medium [35] filtered through 0.22 μm syringe filter (Millipore, USA) prior to use. COC were collected using a stereo zoom microscope (Nikon SMZ1500, Tokyo, Japan) and washed three times in HH medium. The COCs were selected according to their morphological characteristics and graded according to their quality. Only excellent grade oocytes with more than five compact layers of cumulus cells and homogeneous cytoplasm were used for in vitro maturation (IVM) and in vitro fertilization (IVF).

#### In vitro maturation of buffalo oocytes

The oocytes were washed in washing medium 3–4 times (TCM 199 supplemented with 10% fetal bovine serum (FBS), 0.01% sodium pyruvate, 0.005% glutamine and 0.005% streptomycin). Subsequently, they were washed in maturation medium (modified TCM-199 HEPES supplemented with 10% FBS, 0.014% sodium pyruvate, 0.01% glutamine, 0.005% streptomycin, along with 0.5 mg/mL follicle stimulating hormone (FSH), 0.5 mg/mL luteinizing hormone (LH), 1 μg/mL estradiol 17-β, 0.1 mg/mL epidermal growth factor (EGF), 32 mg/mL cysteamine and 50 μg/mL ITS i.e. Insulin-transferrin-sodium selenite media supplement). For IVM, oocytes were placed in drops of 100 μL maturation medium, overlaid with mineral oil and incubated at 38.5 °C in a CO_2_ incubator with 98% humidity and 5% CO_2_ for 24 h.

#### In vitro fertilization process

In vitro fertilization was carried out in 100 μL droplets of Brackett and Oliphant (BO) medium [36] supplemented with 1% BSA (bovine serum albumin) (fatty acid free), 1.9 mg/mL caffeine sodium benzoate, 0.14 mg/mL sodium pyruvate and 0.01 mg/mL heparin. The matured COCs were washed thrice in BO medium prior to their transfer in fertilization drops. The frozen-thawed buffalo semen was processed for in vitro capacitation as per the procedure described earlier [37]. Finally, 50 μL of the processed sperms were added to each droplet containing 20–25 mature COCs, covered with sterile mineral oil and then placed in a CO_2_ incubator with a humidified atmosphere at 38.5 °C in 5% CO_2_ for 14 h.

#### In vitro culture of embryos

Presumptive zygotes were removed from the fertilization drops after 14 h of incubation, adhered cumulus cells were removed by repeated pipetting and washed five times in modified Charles Rosenkrans 2 medium with amino acids (mCR2aa) [38]. After washing, the presumptive zygotes were transferred to 100 μL IVC (in vitro culture) droplets (mCR2aa supplemented with 0.8% BSA, 1.5 mmol/L glucose, 0.0036% sodium pyruvate, 0.014% glutamine, 1% (v/v) MEM (minimal essential medium) nonessential amino acids, 2% (v/v) MEM essential amino acids and 50 μg/mL gentamycin). Zygotes were evaluated for occurrence of cleavage after 48 h of treatment. At 72 h post- treatment, All cleaved embryos were transferred to replacement medium (same as IVC medium except that BSA was replaced with 10% FBS) at 72 h post-treatment and maintained for 8 d at 5% CO_2_ and 38.5 °C with the replacement of medium after every 48 h.

#### Incubation of recombinant and native OVGP1 during IVF

The IVF drops containing the matured oocytes were incubated with recombinant and native buffalo OVGP1 at different concentrations (0, 5, 10 and 20 μg/mL) to assess their effect on in vitro embryo development. In each treatment, 20–25 oocytes were used and the experiments were repeated three times.

### Statistical analysis

The data were analyzed using GraphPad Prism 6 software package (USA). Differences between means were analyzed by Brown-Forsythe test using one- way analysis of variance (ANOVA) procedure. Data were expressed as means ± SEM. Significant differences among means were noted at *P* < 0.05.

## Results

### Purification of recombinant buffalo OVGP1

**Fig. 1:**
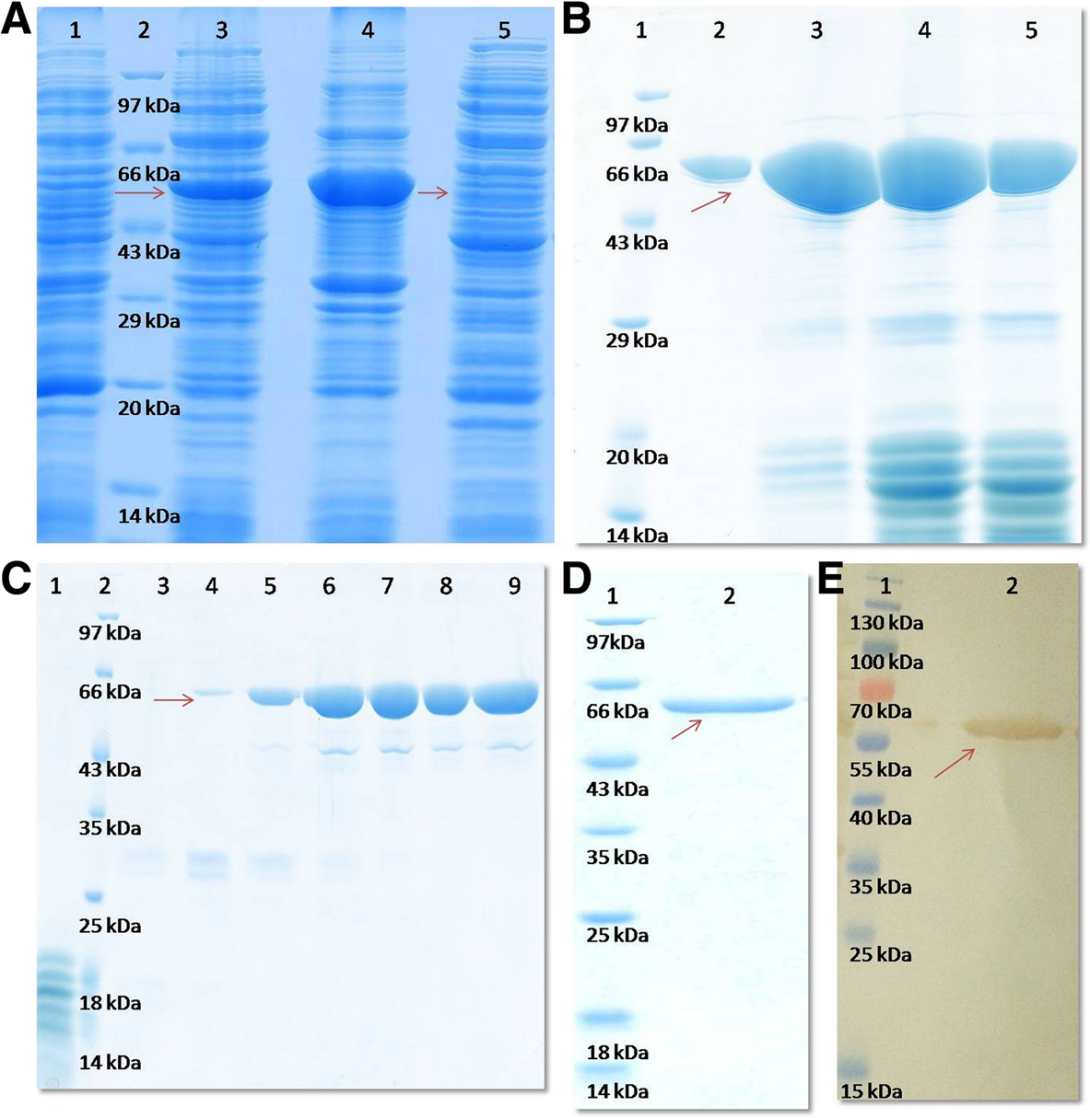
**a** SDS-PAGE analysis showing expression of recombinant OVGP1. Lane 1: Uninduced, lane 2: Molecular weight marker, lane 3: Induced, lane 4: Pellet, lane 5: Soluble fraction; (**b**) SDS-PAGE analysis of His-tag affinity purified recombinant OVGP1 (indicated by *arrow*). Lane 1: Molecular weight marker, lane 2–5: Eluted fractions for partially purified His- tagged recombinant OVGP1; (**c**) Anion- exchange purification of recombinant OVGP1 (indicated by *arrow*). Lane 1: Unbound fraction, lane 2: Molecular weight marker, lane 3–9: Eluted fractions for partially purified His- tagged recombinant OVGP1; and (**d**) Gel filtration purification representing single band (indicated by *arrow*) for purified His- tagged recombinant OVGP1, lane 1: Unstained molecular weight marker, lane 2: purified His- tagged recombinant OVGP1; (**e**) Western blot analysis of purified His-tagged recombinant OVGP1. Lane 1: Prestained molecular weight marker, lane 2: purified His- tagged recombinant OVGP1

**Fig. 2:**
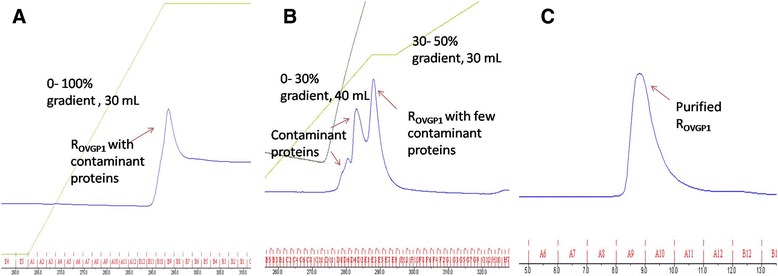
Sequential purification steps of recombinant OVGP1 (R_OVGP1_). Chromatogram of (**a**) IMAC based affinity purification: purification of recombinant OVGP1 through IMAC resulted in a single peak (indicated by *arrow*) after applying a gradient of 0–100% (300 mmol/L imidazole) in 30 mL. The peak fractions contained partially purified recombinant OVGP1. **b** Ion-Exchange Chromatography: Purification step through IEX showing elution of three merged peaks (indicated by *arrows*) during a stepwise gradient run. The latter peak contained recombinant OVGP1 with a few remaining contaminating proteins. **c** Size-Exclusion Chromatography: Purification of recombinant OVGP1 through the step resulted in elution of a peak (indicated by *arrow*) containing purified recombinant OVGP1

The expression of recombinant OVGP1 was observed as a distinct band on SDS- PAGE gel at an expected molecular size of 58 kDa. A major fraction of the protein was expressed as inclusion bodies which are the misfolded protein aggregates. However, a thin band of the soluble fraction of recombinant OVGP1 was also observed at an expected size of 58 kDa (Fig. 1a). Therefore, we were able to purify recombinant OVGP1 from expressed soluble fraction to conduct further experiments for functional characterization. The His-tag affinity chromatography resulted in partial purification of recombinant OVGP1. The SDS-PAGE analysis showed a major band for the partially purified recombinant OVGP1 and some minor bands for the contaminating proteins (Fig. 1b). The ion-exchange chromatography (IEX) step further increased the purity of the protein. However, some non- specific protein bands (~45 kDa & ~ 30 kDa) were still observed on the SDS-PAGE gel (Fig. 1c). The size- exclusion chromatography step resulted in successful purification of OVGP1 to homogeneity and a single band at an expected size of 58 kDa was observed on the SDS-PAGE gel (Fig. 1d). In Western blot analysis, a distinct protein band specific against OVGP1 was examined at an expected size of 58 kDa (Fig. 1e). The chromatograms corresponding to affinity, ion- exchange and size- exclusion chromatography steps are shown in Fig. 2a, b & c, respectively. The identity of purified recombinant OVGP1 was confirmed using mass spectrometer (nLC-ESI-qTof) (Additional file 1).

### Purification of native buffalo OVGP1

**Fig. 3:**
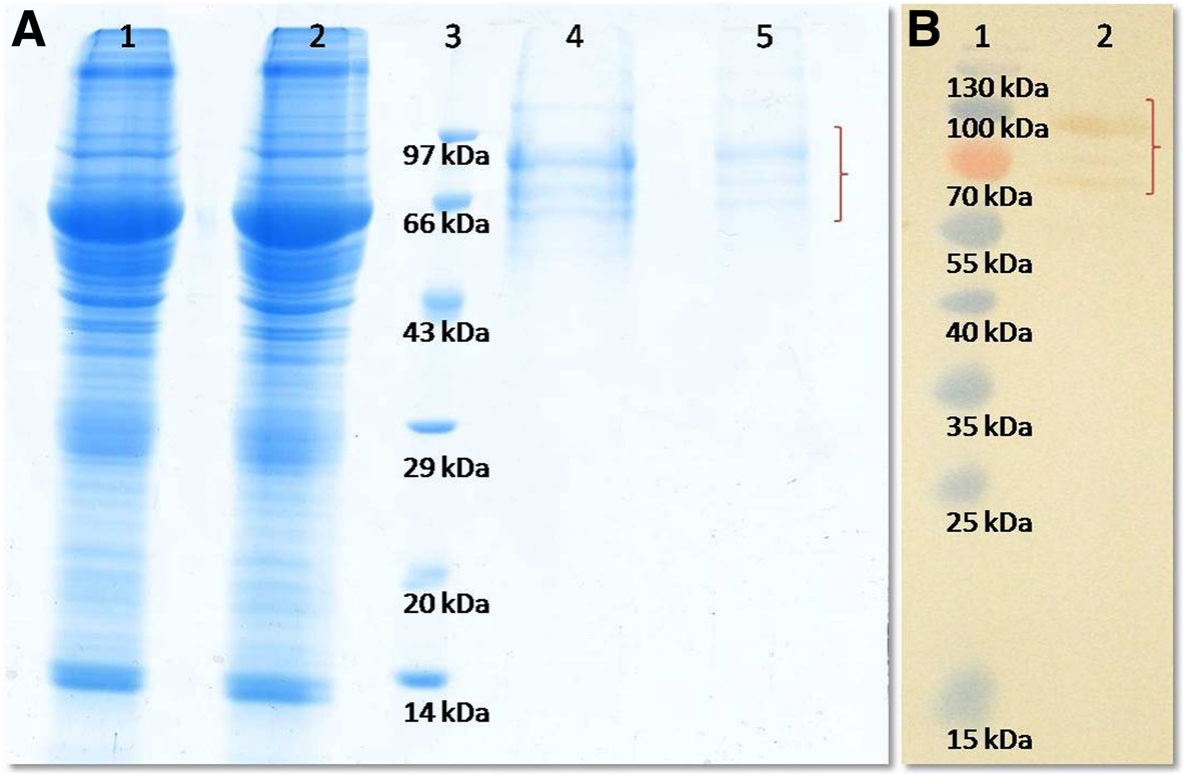
Purification of native OVGP1and its Western blot confirmation: (**a**) Lane 1: Total protein lysate, lane 2: Unbound fraction, lane 3: Molecular weight marker, lane 4: Eluted protein fraction from WGA affinity column, lane 5: Eluted protein fraction from Mono Q anion- exchange column representing three major protein bands (~60- ~95 kDa) for different glycosylated forms of native OVGP1. **b** Western blot confirmation, lane 1: Molecular weight marker, lane 2: Purified native OVGP1

The SDS-PAGE profile of the eluted fractions from WGA column showed multiple protein bands in the range of size ~55 kDa to ~95 kDa (Fig. 3a). The eluted fractions from anion- exchange chromatography step were observed as three major protein bands in the range of size ~60 kDa to ~95 kDa. In Western blot confirmation, three protein bands (~60 kDa to ~95 kDa) were detected with a prominent band at ~95 kDa (Fig. 3b). The identity of the proteins corresponding to the observed bands was confirmed for OVGP1 (Additional file 2) using the mass spectrometer (nLC-ESI-qTof).

### Effect of recombinant and native OVGP1 on sperm characteristics

#### Sperm motility

**Fig. 4:**
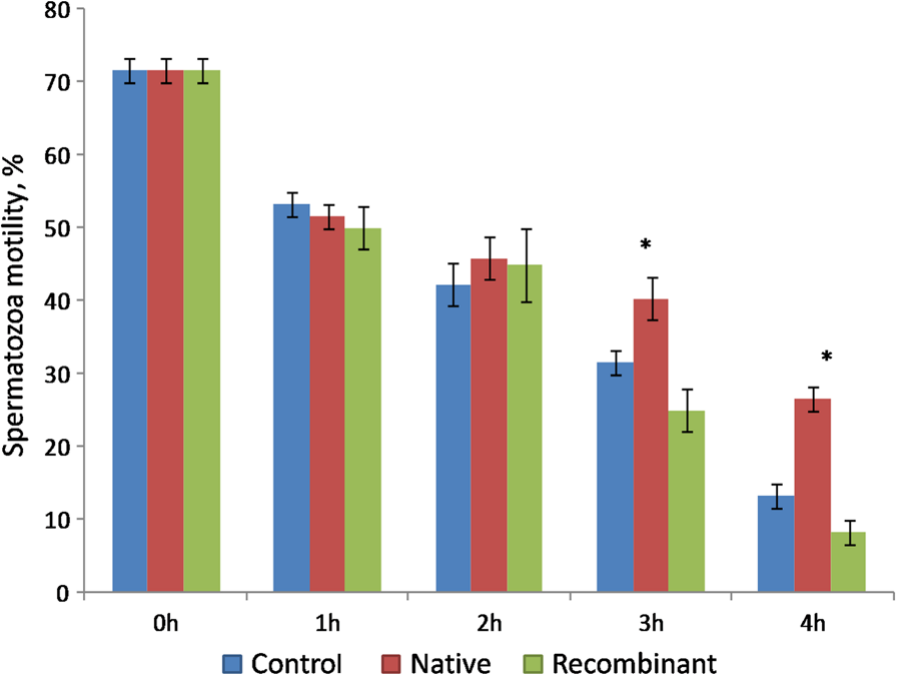
Percentage of motile sperms during incubation with native and recombinant OVGP1as compared to control (without native and recombinant OVGP1). Cryopreserved sperms were incubated with native and recombinant OVGP1 for 4 h at 38 °C, 5% CO_2_. (* significant *P* < 0.05)

There were no significant differences in sperm motility among control, native and recombinant OVGP1-treated groups till 2 h of incubation. However, the proportion of motile sperms at 3 h and 4 h of incubation was greater (*P* < 0.05) in the native OVGP1-treated group as compared to control and recombinant OVGP1-treated groups (Fig. 4).

#### Sperm viability

**Fig. 5:**
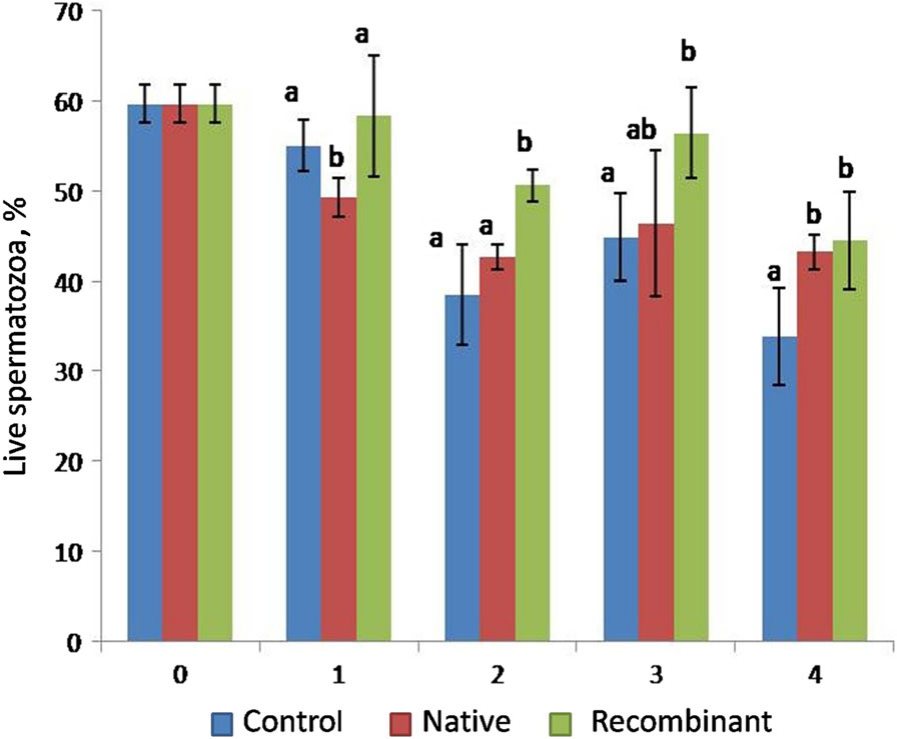
Percentage of live sperms during incubation with native and recombinant OVGP1 as compared to control (without native and recombinant OVGP1). Cryopreserved sperms were incubated with native and recombinant OVGP1 for 4 h at 38 °C, 5% CO_2_. Sperm viability was assessed using SYBR14/PI staining. Superscripts a, b & c indicate the significant differences in the control, native and recombinant OVGP1-treated groups during different hours of incubation (*P* < 0.05)

The effect of recombinant and native OVGP1 on the proportion of viable sperms is shown in Fig. 5. The proportion of viable sperms in control group did not differ significantly with either native or recombinant OVGP1-treated group at 1 h incubation period. The proportion of viable sperms was greater (*P* < 0.05) in the recombinant OVGP1-treated group compared to control at 2 h, 3 h and 4 h of incubation. There were no significant differences in the proportion of viable sperms among control and native OVGP1-treated groups at 2 h and 3 h of incubation.

#### Membrane integrity

**Fig. 6:**
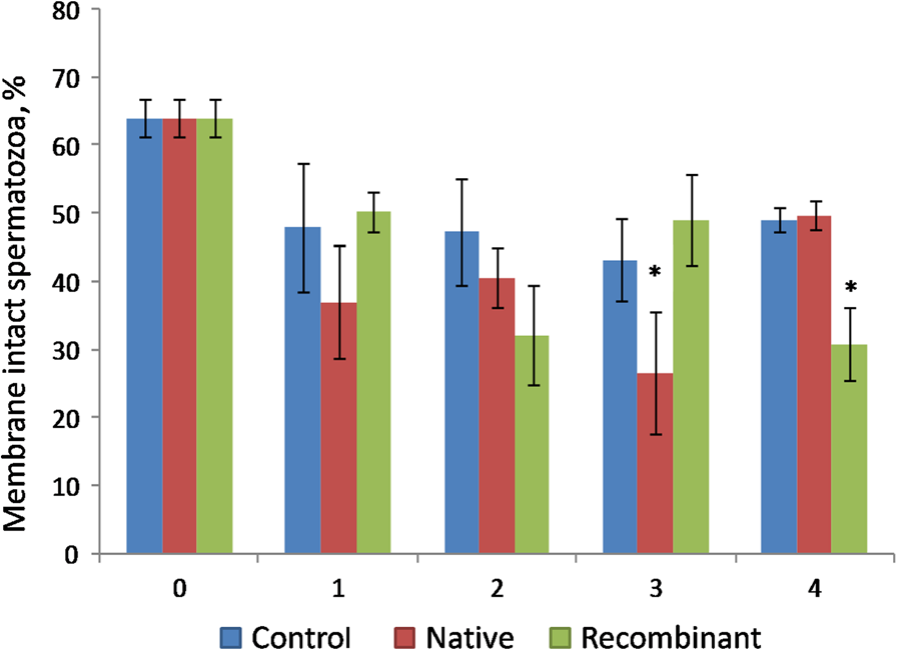
Percentage of membrane intact sperms during incubation with native and recombinant OVGP1 as compared to control (without native and recombinant OVGP1). Cryopreserved sperms were incubated with native and recombinant OVGP1 for 4 h at 38 °C, 5% CO_2_. Membrane integrity was assessed using CFDA/PI staining. (*significant *P* < 0.05)

The effect of recombinant and native OVGP1 on membrane integrity of sperm is shown in Fig. 6. No significant difference was observed in the proportion of membrane intact sperms after treatment with native and recombinant OVGP1 as compared to control at 1 h and 2 h of incubation. The proportion of membrane intact sperms was significantly lower in the native OVGP1-treated group as compared to control and the recombinant OVGP1-treated group at 3 h of incubation. However, both the control and native OVGP1-treated groups had greater (*P* < 0.05) membrane intact sperms in comparison to the recombinant OVGP1-treated group at 4 h of incubation.

#### Capacitation status

**Fig. 7:**
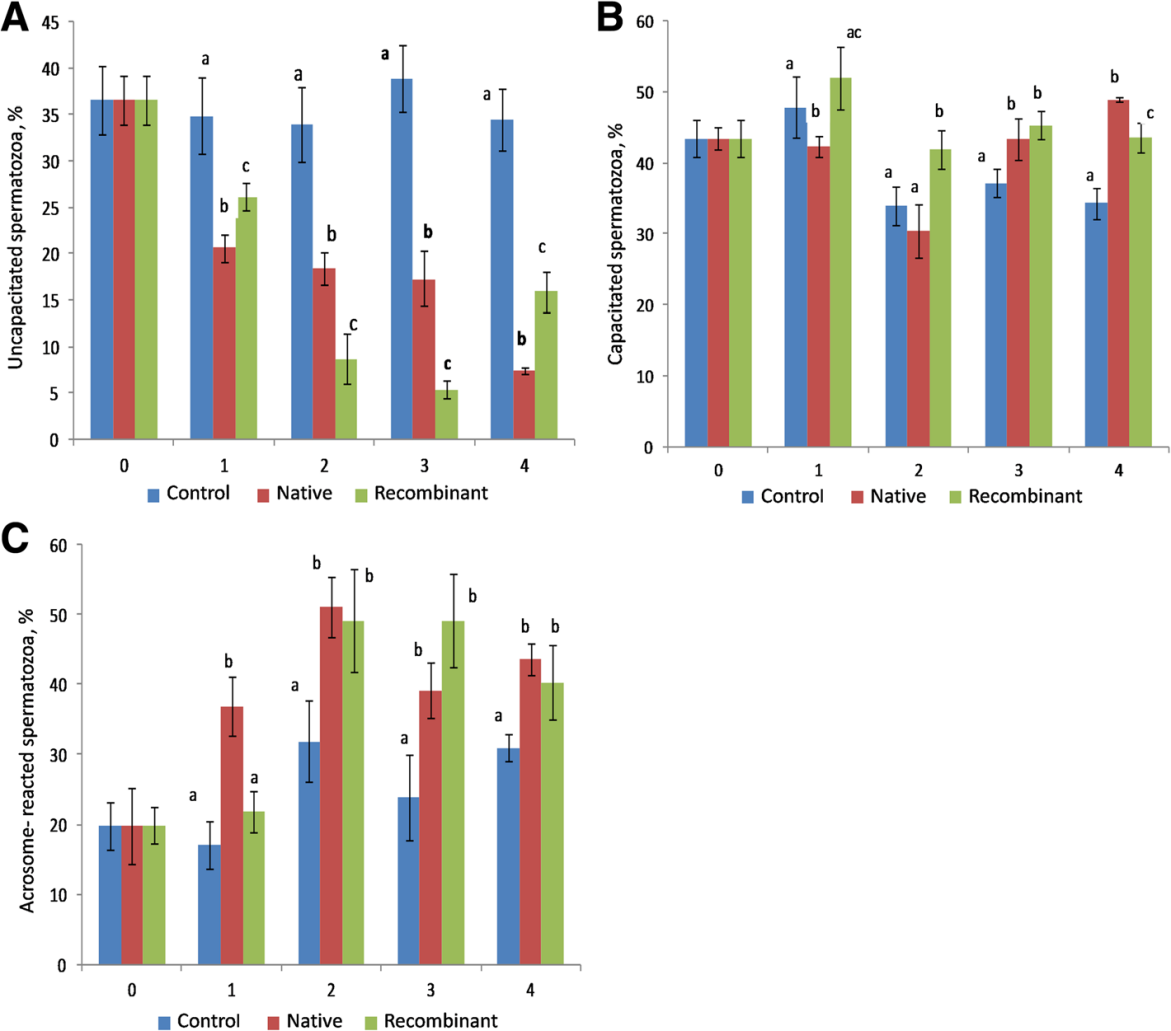
Percentage of uncapacitated (**a**), capacitated (**b**), and acrosome reacted (**c**) sperms during incubation with native and recombinant OVGP1 as compared to control (without native and recombinant OVGP1). Cryopreserved sperms were incubated with native and recombinant OVGP1 for 4 h at 38 °C, 5% CO_2_. Sperm capacitation status was estimated using CTC staining. Superscripts a, b & c indicate the significant differences in the control, native and recombinant OVGP1-treated groups during different hours of incubation (*P* < 0.05)

The proportion of uncapacitated sperms was greater (*P* < 0.05) in control group as compared to native and recombinant OVGP1-treated groups at any given time of incubation from 1 h to 4 h (Fig. 7a). The proportion of capacitated sperms was greater (*P* < 0.05) in control and recombinant OVGP1-treated groups in comparison to the native OVGP1-treated group at 1 h of incubation. The proportion of capacitated sperms was greater (*P* < 0.05) in the recombinant OVGP1-treated group compared to both control and native OVGP1-treated groups at 2 h of incubation. The proportion of capacitated sperms was greater (*P* < 0.05) in both native and recombinant OVGP1-treated groups compared to control group at 3 h and 4 h of incubation (Fig. 7b). The proportion of acrosome-reacted sperms was greater (*P* < 0.05) in the native OVGP1-treated group as compared to control and recombinant OVGP1-treated groups at 1 h of incubation. Native and recombinant OVGP1-treated groups had greater (*P* < 0.05) proportion of acrosome reacted sperms as compared to the control group at 2 h, 3 h and 4 h of incubation (Fig. 7c).

### Effect of recombinant and native OVGP1 on in vitro embryo development

**Fig. 8:**
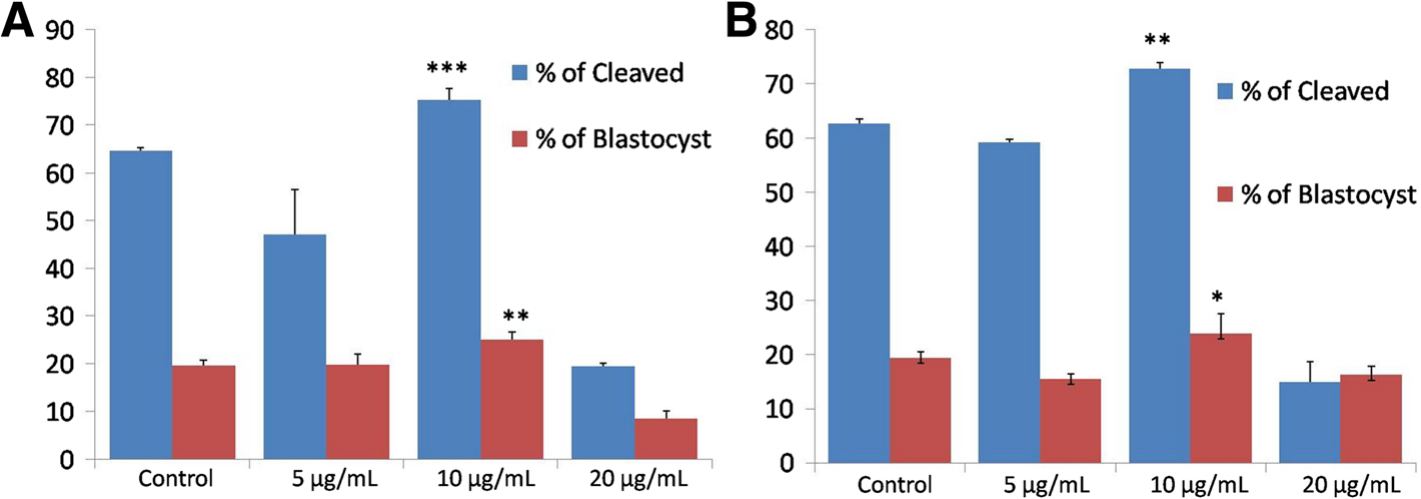
Effect of native (**a**) and recombinant (**b**) OVGP1 on cleavage rate and blastocyst production rate in buffalo. (*, * * and * * * shows the significance of variation among the groups with *P* < 0.05 from three trials of each treatment

The in vitro embryo development was evaluated in the presence of varying concentrations of recombinant and native buffalo OVGP1 i.e. 0, 5, 10 and 20 μg/mL. After in vitro maturation step, native and recombinant OVGP1 were added in IVF drops of their respective groups. In native OVGP1-treated group, the cleavage rates were observed as 64.68%, 47.15%, 75.28% and 19.47% at protein concentrations of 0, 5, 10 and 20 μg/mL, respectively. The rates of blastocysts development after day 7 with respect to the cleaved embryos were observed as 19.65%, 19.77%, 25.07% and 8.55% at 0, 5, 10 and 20 μg/mL of native OVGP1, respectively (Fig. 8a). In recombinant OVGP1-treated group, the cleavage rates were observed as 62.61%, 59.23%, 72.79% and 14.98% at 0, 5, 10 and 20 μg/mL, respectively. The blastocysts development rates were observed to be 19.35%, 15.55%, 23.87% and 16.27% at 0, 5, 10 and 20 μg/mL, respectively in recombinant OVGP1-treated group (Fig. 8b). Therefore, our results show that lower concentration (10 μg/mL) of both native and recombinant OVGP1 confers a positive effect on cleavage rate and blastocyst yield (*P* < 0.05) as compared to higher concentration (20 μg/mL).

## Discussion

The oviduct modulates the sperm functions such as motility, viability, and capacitation with the help of oviduct- specific proteins during fertilization. OVGP1 is one of a major protein that influences various sperm functions and embryo development. The effect of native OVGP1 on sperm functions and IVF is well studied, however, only one report is available on the effect of non- glycosylated recombinant OVGP1 on sperm binding and IVF [39]. In the present study, we have performed a comparative assessment of the both native and recombinant OVGP1 (expressed in *E. coli*) on various sperm functions and in vitro embryo development.

In the female reproductive tract, oviductal fluid secreted by the oviduct interacts with sperms to modulate their functions and provides an optimal environment for the ovulated oocyte. Various research groups have reported that many proteins are secreted from the oviduct, which promote the viability of sperm [40, 41], prolong sperm motility [42], protect membrane integrity [43] and induce sperm capacitation [22]. It has been reported that when semen samples were incubated in vitro at 37 °C for an hour, their quality declined drastically in buffalo [44]. In contrast, sperms can survive for more than 20 h in the oviduct in cattle [45]. We observed enhanced motility of sperm in the presence of native OVGP1 as compared to the control and recombinant OVGP1 at 3 h and 4 h of incubation, although there was a progressive decrease in the percentage of motile sperms in both recombinant and native OVGP1-treated groups. However, the proportion of viable sperms was significantly greater in the recombinant OVGP1-treated group compared to the native OVGP1-treated group during 2 h, 3 h, and 4 h of the incubation period. The percentage of membrane intact sperms decreased significantly in the recombinant OVGP1-treated group compared to the native OVGP1 and control at 4 h of incubation time. Also, the percentage of capacitated and acrosome reacted sperms was significantly lower in the recombinant OVGP1-treated group as compared to the native OVGP1-treated group at 4 h of incubation time. Our findings suggest that optimal motility, viability, sperm integrity, capacitation and acrosome reaction of sperms are enhanced upon treatment with OVGP1 (both native and recombinant). These findings suggest that OVGP1 interacts with the sperm surface to perform these functions. However, the reduction in the motility of sperm after 3 h and 4 h; and membrane integrity and capacitation after 4 h of inoculation, in the presence of non-glycosylated recombinant OVGP1 suggests that glycan moieties in the native OVPG1 may better support these sperm parameters. In the golden hamster, it was reported that OVGP1 has a higher affinity to the acrosomal cap and the plasma membrane of the tail leading to an increase in motility and acrosome integrity [46]. Recombinant glycosylated form of hamster OVGP1, expressed in human embryonic kidney 293 (HEK293) cells, was shown to enhance the tyrosine phosphorylation of sperm proteins and acrosome reaction. Further, it was reported that sperm-oocyte binding was also improved upon pre-treatment of oocytes and sperm with recombinant hamster OVGP1 [47]. Similar observations were reported for recombinant glycosylated human OVGP1 expressed in HEK293 cells where tyrosine phosphorylation of sperm proteins was enhanced, and the number of acrosome-reacted sperms was significantly increased [48]. It was reported that glycosylation differences detected in the estrous cycle could be responsible for a different biological role of the oviductal-secreted glycoproteins in golden hamster [49, 50]. In the present study, the recombinant OVGP1 expressed in *E. coli* lacks the carbohydrate moieties, and this may be responsible for the variations observed in the effects of native and recombinant OVGP1 on different parameters of sperms i.e. motility, membrane integrity and capacitation status in later hours of incubation.

In the IVF experiments, cleavage and blastocyst development rates were increased after exposure to recombinant and native buffalo OVGP1. However, we did not find any significant difference in the percentage of cleavage and blastocyst development between glycosylated native and non- glycosylated recombinant OVGP1. It has been reported that recombinant feline OVGP1 expressed in *E. coli* enhances the binding of sperms to oocyte zona pellucida and also improves the quality of embryos without any significant effect on cleavage and blastocyst development [39]. It has been proposed that OVGP1 exerts its effect by binding to the glycan moieties of glycoproteins located on ZP of oocytes and some specific regions of sperm [1]. OVGP1 decreases polyspermy increases the rate of in vitro maturation, fertilization and embryo development in many species [2]. Studies have also demonstrated that incubation of oocytes with bovine oviductal fluid resulted in a hardening of zona pellucida [51]. Similar observations were made when purified goat OVGP1 treatment was given to oocytes and a reduction in the time of the dissolution of zona pellucida was observed [18]. Therefore, OVGP1 enhances the fertilization rates possibly by protecting the integrity of the zona pellucida from oviductal proteases inside the lumen of oviduct and blocks polyspermy. In our study, the recombinant OVGP1 expressed in *E. coli* lacks the carbohydrate moieties; however, the chitinase- like domain present in N- terminal region retains its sugar binding properties that may interact with the gametes. Therefore, it can be interpreted that OVGP1 lacking carbohydrate moieties still retains some properties of glycosylated native OVGP1. This could be a possible explanation for an increase in the cleavage and blastocyst development rate in recombinant OVGP1 in comparison to the control and a slightly decreased rate as compared to the native OVGP1.

In the present study, the IVF experiments were performed using frozen- thawed sperms. However, in a previous study [52], an increase in the IVF efficiency was observed when fresh sperms were used as compared to the frozen- thawed in response to the porcine oviductal fluid. Therefore, it can be interpreted that IVF efficiency in buffalo may vary when using a different source of sperm i.e. fresh semen. Further, in contrary to our present study, previous studies involved the use of oviductal fluid possessing the total proteins including OVGP1 which may also be considered to influence the IVF efficiency. Furthermore, in our experimental procedures during IVF, the effect of OVGP1 (either native or recombinant) was found to be dose-dependent. The increased rates of cleavage and blastocysts development were observed at 10 μg/mL in comparison to the control. The native and recombinant OVGP1 significantly decreased the cleavage and blastocyst development rates at higher concentration (20 μg/mL). pOSP (porcine oviduct- specific glycoprotein) at 50 and 100 μg/mL supplemented during IVC had an adverse effect on the number of embryos that developed to blastocysts [53]. Similar observations were made in the goat OVGP1 where a lower concentration (10 μg/mL) increased the cleavage rate, morula and blastocyst yield significantly as compared to a higher concentration (100 μg/mL) [18]. This report suggests that the use of an optimum concentration of OVGP1 is an important parameter for in vitro embryo development because higher concentrations exhibit an inhibitory effect.

## Conclusions

In the present study, both recombinant and native buffalo OVGP1 were observed to impart a significant beneficial effect on sperm features and in vitro embryo development. Taken together, the results of the present study indicate the possibility of using buffalo OVGP1 as a medium supplement to improve the sperm quality and in vitro blastocysts development rates in buffalo.

## Additional files

Additional file 1: Identification of recombinant OVGP1 by LC MS/MS. (PDF 121 kb)

Additional file 2: Identification of native OVGP1 by LC MS/MS. (PDF 182 kb)
