# Supplementary material for: Effect of recombinant and native buffalo OVGP1 on sperm functions and in vitro embryo development: a comparative study
Source: J Anim Sci Biotechnol. 2017 Sep 1;8:69. doi: 10.1186/s40104-017-0201-5 (PMC5580196; doi:10.1186/s40104-017-0201-5)
Supplement: Supplementary file 1 — Identification of recombinant OVGP1 by LC MS/MS. (PDF 121 kb) [file 40104_2017_201_MOESM1_ESM.pdf]

MATRIX SCIENCE MASCOT Search Results

Protein View: tr|A1L579|A1L579_BOVIN

Oviduct-specific glycoprotein OS=Bos taurus GN=OVGP1 PE=2 SV=1

**Database:**	Bostaurus_uniprot
**Score:**	231
**Nominal mass (M_r):**	53486
**Calculated pI:**	9.61

Sequence similarity is available as **an NCBI BLAST search of tr|A1L579|A1L579_BOVIN against nr**.

Search parameters

**MS data file:**	DATA.TXT
**Enzyme:**	Trypsin: cuts C-term side of KR unless next residue is P.
**Fixed modifications:**	**Carbamidomethyl (C)**
**Variable modifications:**	**Oxidation (M)**

Protein sequence coverage: 26%

Matched peptides shown in ***bold red***.

1 MSNNQIVPKD PQDEK**ILYPE** **FNK**LKERNRG LKTLLSIGGW NFGTVR**FTTM**
51 **LSTFSNR**ERF VSSVIALLR**T** **HGFDGLDLFF** **LYPGLR**GSPA RDRWTFVFLL
101 EELLQAFKNE AQLTMRPRLL LSAAVSGDPH VVQKAYEARL LGRLLDFISV
151 LSYDLHGSWE K**VTGHNSPLF** **SLPGDPKSSA** **YAMNYWR**QLG VPPEK**LLMGL**
201 **PTYGR**TFHLL KASQNELRAQ AVGPASPGKY TKQAGFLAYY EICCFVRRAK
251 KR**WINDQYVP** **YAFK**GK**EWVG** **YDDAISFGYK** AFFIKR**EHFG** **GAMVWTLDLD**
301 **DFR**GYFCGTG PFPLVHTLNN LLVNDEFSST PSPK**FWFSTA** **VNSSR**IGPEM
351 PTMTRDLTTG LGILPPGGEA VATETHRKSE TMTITPKGEI ATPTRTPLSF
401 GRRTAAPEGK TESPGEKPLT TVGHLAVSPG GIAVGPVRLQ TGQKVTPPGR
451 KAGVPEKVTT PSGKMTVTPD GRAETLERRL

Unformatted sequence string: **480 residues** (for pasting into other applications).

Sort peptides by ◉ Residue Number ○ Increasing Mass ○ Decreasing Mass

Show predicted peptides also

Query	Start – End	Observed	Mr(expt)	Mr(calc)	ppm	M	Score	Expect	Rank	U	Peptide
2887	16 – 23	512.2920	1022.5693	1022.5437	25.1	0	7	9.9	1	U	K.ILYPEFNK.L
2888	16 – 23	512.2920	1022.5694	1022.5437	25.2	0	3	23	2	U	K.ILYPEFNK.L
4425	47 – 57	660.8318	1319.6491	1319.6180	23.5	0	36	0.015	1	U	R.FTTMLSTFSNR.E + Oxidation (M)
4426	47 – 57	660.8321	1319.6496	1319.6180	24.0	0	14	2.4	1	U	R.FTTMLSTFSNR.E + Oxidation (M)
6514	70 – 86	656.6860	1967.0361	1966.9942	21.3	0	17	0.67	1	U	R.THGFDGLDLFFLYPGLR.G
6515	70 – 86	656.6860	1967.0363	1966.9942	21.4	0	29	0.038	1	U	R.THGFDGLDLFFLYPGLR.G
5205	162 – 177	555.9711	1664.8914	1664.8522	23.6	0	15	1.4	1	U	K.VTGHNSPLFSLPGDPK.S
5206	162 – 177	555.9714	1664.8923	1664.8522	24.0	0	13	2.1	1	U	K.VTGHNSPLFSLPGDPK.S
4273	178 – 187	632.7883	1263.5620	1263.5342	22.0	0	4	10	1	U	K.SSAYAMNYWR.Q + Oxidation (M)
3605	196 – 205	560.8262	1119.6378	1119.6110	23.9	0	48	0.00059	1	U	K.LLMGLPTYGR.T
3606	196 – 205	560.8266	1119.6386	1119.6110	24.7	0	19	0.43	1	U	K.LLMGLPTYGR.T
3642	196 – 205	568.8234	1135.6323	1135.6060	23.2	0	8	5.6	1	U	K.LLMGLPTYGR.T + Oxidation (M)
3643	196 – 205	568.8234	1135.6323	1135.6060	23.2	0	12	2.3	1	U	K.LLMGLPTYGR.T + Oxidation (M)
4868	253 – 264	772.3991	1542.7837	1542.7507	21.4	0	25	0.16	1	U	R.WINDQYVPYAFK.G
4869	253 – 264	772.3991	1542.7837	1542.7507	21.4	0	16	1.4	1	U	R.WINDQYVPYAFK.G
5172	267 – 280	825.3943	1648.7741	1648.7409	20.1	0	35	0.017	1	U	K.EWVGYDDAISFGYK.A
5173	267 – 280	825.3951	1648.7757	1648.7409	21.1	0	54	0.00019	1	U	K.EWVGYDDAISFGYK.A
6752	287 – 303	670.3272	2007.9599	2007.9149	22.4	0	29	0.045	1	U	R.EHFGGAMVWTLDLDDFR.G
6753	287 – 303	670.3276	2007.9609	2007.9149	22.9	0	18	0.62	1	U	R.EHFGGAMVWTLDLDDFR.G
6878	287 – 303	675.6588	2023.9546	2023.9098	22.1	0	33	0.028	1	U	R.EHFGGAMVWTLDLDDFR.G + Oxidation (M)
6879	287 – 303	675.6590	2023.9550	2023.9098	22.3	0	33	0.027	1	U	R.EHFGGAMVWTLDLDDFR.G + Oxidation (M)
4346	335 – 345	651.3326	1300.6506	1300.6200	23.5	0	29	0.067	1	U	K.FWFSTAVNSSR.I
4347	335 – 345	651.3331	1300.6516	1300.6200	24.3	0	8	8.2	1	U	K.FWFSTAVNSSR.I

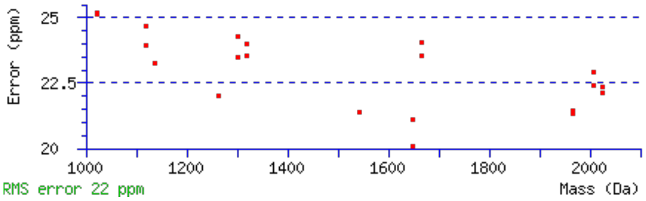

**Mascot:** http://www.matrixscience.com/
