# Supplementary material for: Effect of recombinant and native buffalo OVGP1 on sperm functions and in vitro embryo development: a comparative study
Source: J Anim Sci Biotechnol. 2017 Sep 1;8:69. doi: 10.1186/s40104-017-0201-5 (PMC5580196; doi:10.1186/s40104-017-0201-5)
Supplement: Supplementary file 2 — Identification of native OVGP1 by LC MS/MS. (PDF 182 kb) [file 40104_2017_201_MOESM2_ESM.pdf]

MATRIX SCIENCE MASCOT Search Results

Protein View: tr|A1L579|A1L579_BOVIN

Oviduct-specific glycoprotein OS=Bos taurus GN=OVGP1 PE=2 SV=1

**Database:**	Bostaurus_uniprot
**Score:**	2126
**Nominal mass (M_r):**	53486
**Calculated pI:**	9.61

Sequence similarity is available as **an NCBI BLAST search of tr|A1L579|A1L579_BOVIN against nr**.

Search parameters

**MS data file:**	DATA.TXT
**Enzyme:**	Trypsin: cuts C-term side of KR unless next residue is P.
**Fixed modifications:**	**Carbamidomethyl (C)**
**Variable modifications:**	**Oxidation (M)**

Protein sequence coverage: 29%

Matched peptides shown in ***bold red***.

1 MSNNQIVPKD PQDEK**ILYPE** **FNK**LKERNRG LKTLLSIGGW NFGTVR**FTTM**
51 **LSTFSNR**ERF VSSVIALLRT HGFDGLDLFF LYPGLRGSPA RDRWTFVFLL
101 EELLQAFK**NE** **AQLTMRPR**LL LSAAVSGDPH VVQKAYEARL LGRLLDFISV
151 LSYDLHGSWE K**VTGHNSPLF** **SLPGDPKSSA** **YAMNYWRQLG** **VPPEKLLMGL**
201 **PTYGR**TFHLL K**ASQNELR**AQ AVGPASPGKY TKQAGFLAYY EICCFVRRAK
251 KR**WINDQYVP** **YAFKGKEWVG** **YDDAISFGYK** **AFFIK**R**EHFG** **GAMVWTLDLD**
301 **DFR**GYFCGTG PFPLVHTLNN LLVNDEFSST PSPKFWFSTA VNSSR**IGPEM**
351 **PTMTR**DLTTG LGILPPGGEA VATETHRKSE TMTITPKGEI ATPTRTPLSF
401 GRRTAAPEGK TESPGEKPLT TVGHLAVSPG GIAVGPVRLQ TGQKVTPPGR
451 KAGVPEKVTT PSGKMTVTPD GRAETLERRL

Unformatted sequence string: **480 residues** (for pasting into other applications).

Sort peptides by ◉ Residue Number ○ Increasing Mass ○ Decreasing Mass

Show predicted peptides also

Query	Start – End	Observed	Mr(expt)	Mr(calc)	ppm	M	Score	Expect	Rank	U	Peptide
998	16 – 23	512.2857	1022.5568	1022.5437	12.9	0	22	0.3	1		K.ILYPEFNK.L
999	16 – 23	512.2858	1022.5570	1022.5437	13.1	0	27	0.11	1		K.ILYPEFNK.L
1000	16 – 23	512.2858	1022.5570	1022.5437	13.1	0	28	0.089	1		K.ILYPEFNK.L
1001	16 – 23	512.2861	1022.5577	1022.5437	13.7	0	23	0.28	1		K.ILYPEFNK.L
1002	16 – 23	512.2862	1022.5578	1022.5437	13.9	0	13	2.4	1		K.ILYPEFNK.L
1003	16 – 23	512.2862	1022.5579	1022.5437	13.9	0	13	2.5	1		K.ILYPEFNK.L
1004	16 – 23	512.2864	1022.5583	1022.5437	14.3	0	14	1.9	1		K.ILYPEFNK.L
1005	16 – 23	512.2866	1022.5587	1022.5437	14.7	0	20	0.49	1		K.ILYPEFNK.L
1006	16 – 23	512.2867	1022.5589	1022.5437	14.9	0	20	0.52	1		K.ILYPEFNK.L
1487	47 – 57	652.8269	1303.6392	1303.6231	12.4	0	53	0.00035	1	U	R.FTTMLSTFSNR.E
1488	47 – 57	652.8278	1303.6410	1303.6231	13.8	0	1	49	1	U	R.FTTMLSTFSNR.E
1489	47 – 57	652.8280	1303.6415	1303.6231	14.2	0	1	51	1	U	R.FTTMLSTFSNR.E
1491	47 – 57	652.8291	1303.6436	1303.6231	15.7	0	46	0.0015	1	U	R.FTTMLSTFSNR.E
1492	47 – 57	652.8293	1303.6441	1303.6231	16.1	0	1	51	1	U	R.FTTMLSTFSNR.E
1502	47 – 57	660.8246	1319.6347	1319.6180	12.7	0	51	0.00044	1	U	R.FTTMLSTFSNR.E + Oxidation (M)
1503	47 – 57	660.8247	1319.6348	1319.6180	12.8	0	42	0.003	1	U	R.FTTMLSTFSNR.E + Oxidation (M)
1504	47 – 57	660.8247	1319.6349	1319.6180	12.8	0	47	0.00099	1	U	R.FTTMLSTFSNR.E + Oxidation (M)
1505	47 – 57	660.8248	1319.6351	1319.6180	13.0	0	61	4.2e-005	1	U	R.FTTMLSTFSNR.E + Oxidation (M)
1506	47 – 57	660.8248	1319.6351	1319.6180	13.0	0	58	9e-005	1	U	R.FTTMLSTFSNR.E + Oxidation (M)
1507	47 – 57	660.8248	1319.6351	1319.6180	13.0	0	67	9.3e-006	1	U	R.FTTMLSTFSNR.E + Oxidation (M)
1508	47 – 57	660.8248	1319.6351	1319.6180	13.0	0	56	0.00013	1	U	R.FTTMLSTFSNR.E + Oxidation (M)
1509	47 – 57	660.8249	1319.6353	1319.6180	13.1	0	28	0.08	1	U	R.FTTMLSTFSNR.E + Oxidation (M)
1510	47 – 57	660.8252	1319.6358	1319.6180	13.5	0	41	0.004	1	U	

Query	Start - End	Observed	Mr(expt)	Mr(calc)	ppm	M	Score	Expect	Rank	U	Peptide
											R.FTTMLSTFSNR.E + Oxidation (M)
1511	47 - 57	660.8254	1319.6362	1319.6180	13.8	0	42	0.0036	1	U	R.FTTMLSTFSNR.E + Oxidation (M)
1512	47 - 57	660.8258	1319.6371	1319.6180	14.5	0	33	0.026	1	U	R.FTTMLSTFSNR.E + Oxidation (M)
1513	47 - 57	660.8261	1319.6377	1319.6180	14.9	0	4	21	1	U	R.FTTMLSTFSNR.E + Oxidation (M)
1384	109 - 118	411.2185	1230.6336	1230.6139	16.1	0	16	2.4	1		K.NEAQLTMRPR.L + Oxidation (M)
1731	162 - 177	555.9654	1664.8743	1664.8522	13.2	0	34	0.019	1		K.VTGHNSPLFSLPGDPK.S
1732	162 - 177	555.9654	1664.8743	1664.8522	13.2	0	23	0.24	1		K.VTGHNSPLFSLPGDPK.S
1733	162 - 177	555.9654	1664.8744	1664.8522	13.3	0	40	0.0042	1		K.VTGHNSPLFSLPGDPK.S
1734	162 - 177	555.9654	1664.8744	1664.8522	13.3	0	21	0.39	1		K.VTGHNSPLFSLPGDPK.S
1735	162 - 177	555.9655	1664.8747	1664.8522	13.5	0	32	0.03	1		K.VTGHNSPLFSLPGDPK.S
1736	162 - 177	555.9656	1664.8748	1664.8522	13.6	0	24	0.18	1		K.VTGHNSPLFSLPGDPK.S
1737	162 - 177	555.9657	1664.8752	1664.8522	13.8	0	12	3.1	1		K.VTGHNSPLFSLPGDPK.S
1738	162 - 177	833.4454	1664.8763	1664.8522	14.5	0	2	26	1		K.VTGHNSPLFSLPGDPK.S
1739	162 - 177	555.9662	1664.8767	1664.8522	14.7	0	3	24	2		K.VTGHNSPLFSLPGDPK.S
1740	162 - 177	555.9662	1664.8768	1664.8522	14.7	0	3	23	1		K.VTGHNSPLFSLPGDPK.S
1742	162 - 177	555.9663	1664.8771	1664.8522	14.9	0	25	0.13	1		K.VTGHNSPLFSLPGDPK.S
1743	162 - 177	555.9669	1664.8788	1664.8522	16.0	0	8	7.9	1		K.VTGHNSPLFSLPGDPK.S
1744	162 - 177	833.4468	1664.8791	1664.8522	16.1	0	11	3.2	1		K.VTGHNSPLFSLPGDPK.S
1414	178 - 187	624.7847	1247.5549	1247.5393	12.5	0	56	8.4e-005	1		K.SSAYAMNYWR.Q
1415	178 - 187	624.7848	1247.5550	1247.5393	12.6	0	17	0.72	1		K.SSAYAMNYWR.Q
1416	178 - 187	624.7848	1247.5551	1247.5393	12.7	0	62	2e-005	1		K.SSAYAMNYWR.Q
1417	178 - 187	624.7848	1247.5551	1247.5393	12.7	0	44	0.0013	1		K.SSAYAMNYWR.Q
1418	178 - 187	624.7849	1247.5553	1247.5393	12.9	0	26	0.093	1		K.SSAYAMNYWR.Q
1419	178 - 187	624.7852	1247.5557	1247.5393	13.2	0	52	0.00025	1		K.SSAYAMNYWR.Q
1420	178 - 187	624.7852	1247.5559	1247.5393	13.3	0	40	0.0038	1		K.SSAYAMNYWR.Q
1421	178 - 187	624.7853	1247.5561	1247.5393	13.5	0	44	0.0015	1		K.SSAYAMNYWR.Q
1422	178 - 187	624.7860	1247.5574	1247.5393	14.5	0	16	1	1		K.SSAYAMNYWR.Q
1444	178 - 187	632.7823	1263.5500	1263.5342	12.5	0	54	9e-005	1		K.SSAYAMNYWR.Q + Oxidation (M)
1445	178 - 187	632.7824	1263.5502	1263.5342	12.7	0	36	0.0055	1		K.SSAYAMNYWR.Q + Oxidation (M)
1446	178 - 187	632.7825	1263.5503	1263.5342	12.8	0	37	0.0048	1		K.SSAYAMNYWR.Q + Oxidation (M)
1447	178 - 187	632.7829	1263.5512	1263.5342	13.4	0	50	0.00023	1		K.SSAYAMNYWR.Q + Oxidation (M)
1448	178 - 187	632.7830	1263.5513	1263.5342	13.6	0	3	12	1		K.SSAYAMNYWR.Q + Oxidation (M)
1449	178 - 187	632.7830	1263.5515	1263.5342	13.7	0	38	0.0037	1		K.SSAYAMNYWR.Q + Oxidation (M)
1450	178 - 187	632.7832	1263.5518	1263.5342	13.9	0	16	0.6	1		K.SSAYAMNYWR.Q + Oxidation (M)
1451	178 - 187	632.7833	1263.5521	1263.5342	14.1	0	11	1.8	1		K.SSAYAMNYWR.Q + Oxidation (M)
1452	178 - 187	632.7835	1263.5524	1263.5342	14.4	0	33	0.011	1		K.SSAYAMNYWR.Q + Oxidation (M)
1453	178 - 187	632.7840	1263.5533	1263.5342	15.1	0	10	2.4	1		K.SSAYAMNYWR.Q + Oxidation (M)
1456	178 - 187	632.7845	1263.5544	1263.5342	16.0	0	18	0.43	1		K.SSAYAMNYWR.Q + Oxidation (M)
640	188 - 195	434.2570	866.4995	866.4862	15.4	0	12	1.6	1		R.QLGVPPEK.L
641	188 - 195	434.2571	866.4997	866.4862	15.6	0	22	0.18	1		R.QLGVPPEK.L
642	188 - 195	434.2571	866.4997	866.4862	15.6	0	22	0.17	1		R.QLGVPPEK.L
643	188 - 195	434.2574	866.5002	866.4862	16.2	0	13	1.4	1		R.QLGVPPEK.L
644	188 - 195	434.2574	866.5002	866.4862	16.2	0	10	2.8	1		R.QLGVPPEK.L
645	188 - 195	434.2575	866.5004	866.4862	16.4	0	15	0.92	1		R.QLGVPPEK.L
646	188 - 195	434.2576	866.5006	866.4862	16.7	0	21	0.2	1		R.QLGVPPEK.L
647	188 - 195	434.2576	866.5007	866.4862	16.8	0	14	1	1		R.QLGVPPEK.L
648	188 - 195	434.2576	866.5007	866.4862	16.8	0	6	6.9	1		R.QLGVPPEK.L
649	188 - 195	434.2577	866.5009	866.4862	17.0	0	13	1.2	1		R.QLGVPPEK.L
650	188 - 195	434.2578	866.5010	866.4862	17.1	0	17	0.5	1		R.QLGVPPEK.L
651	188 - 195	434.2579	866.5013	866.4862	17.4	0	21	0.19	1		R.QLGVPPEK.L
652	188 - 195	434.2581	866.5016	866.4862	17.8	0	12	1.8	1		R.QLGVPPEK.L
653	188 - 195	434.2581	866.5017	866.4862	17.9	0	9	3.1	1		R.QLGVPPEK.L
654	188 - 195	434.2586	866.5026	866.4862	19.0	0	6	7.4	1		R.QLGVPPEK.L
1103	196 - 205	560.8199	1119.6253	1119.6110	12.8	0	38	0.0067	1	U	K.LLMGLPTYGR.T
1104	196 - 205	560.8203	1119.6261	1119.6110	13.4	0	41	0.0033	1	U	K.LLMGLPTYGR.T
1105	196 - 205	560.8205	1119.6264	1119.6110	13.7	0	28	0.075	1	U	K.LLMGLPTYGR.T
1106	196 - 205	560.8205	1119.6265	1119.6110	13.8	0	19	0.56	1	U	K.LLMGLPTYGR.T
1107	196 - 205	560.8206	1119.6266	1119.6110	13.9	0	5	15	1	U	K.LLMGLPTYGR.T
1108	196 - 205	560.8206	1119.6267	1119.6110	14.0	0	23	0.2	1	U	K.LLMGLPTYGR.T
1109	196 - 205	560.8207	1119.6268	1119.6110	14.1	0	15	1.5	1	U	K.LLMGLPTYGR.T
1110	196 - 205	560.8210	1119.6275	1119.6110	14.7	0	13	2	1	U	K.LLMGLPTYGR.T
1111	196 - 205	560.8211	1119.6276	1119.6110	14.8	0	6	11	1	U	K.LLMGLPTYGR.T

Query	Start - End	Observed	Mr(expt)	Mr(calc)	ppm	M	Score	Expect	Rank	U	Peptide
1112	196 - 205	560.8217	1119.6288	1119.6110	15.9	0	2	28	1	U	K.LLMGLPTYGR.T
1113	196 - 205	560.8218	1119.6290	1119.6110	16.1	0	12	2.8	1	U	K.LLMGLPTYGR.T
1114	196 - 205	560.8218	1119.6291	1119.6110	16.1	0	19	0.54	1	U	K.LLMGLPTYGR.T
1115	196 - 205	560.8220	1119.6293	1119.6110	16.4	0	20	0.43	1	U	K.LLMGLPTYGR.T
1244	196 - 205	568.8175	1135.6205	1135.6060	12.8	0	50	0.00045	1	U	K.LLMGLPTYGR.T + Oxidation (M)
1245	196 - 205	568.8176	1135.6206	1135.6060	12.9	0	35	0.013	1	U	K.LLMGLPTYGR.T + Oxidation (M)
1247	196 - 205	568.8177	1135.6209	1135.6060	13.2	0	49	0.00061	1	U	K.LLMGLPTYGR.T + Oxidation (M)
1248	196 - 205	568.8178	1135.6210	1135.6060	13.2	0	41	0.0038	1	U	K.LLMGLPTYGR.T + Oxidation (M)
1249	196 - 205	568.8179	1135.6211	1135.6060	13.4	0	37	0.0088	1	U	K.LLMGLPTYGR.T + Oxidation (M)
557	212 - 218	409.2183	816.4221	816.4090	16.1	0	28	0.11	1		K.ASQNELR.A
558	212 - 218	409.2186	816.4226	816.4090	16.7	0	47	0.0015	1		K.ASQNELR.A
1636	253 - 264	772.3913	1542.7681	1542.7507	11.3	0	0	47	3	U	R.WINDQYVPYAFK.G
1637	253 - 264	772.3916	1542.7685	1542.7507	11.6	0	27	0.099	1	U	R.WINDQYVPYAFK.G
1638	253 - 264	772.3916	1542.7687	1542.7507	11.7	0	68	8.4e-006	1	U	R.WINDQYVPYAFK.G
1639	253 - 264	772.3917	1542.7688	1542.7507	11.7	0	48	0.00082	1	U	R.WINDQYVPYAFK.G
1640	253 - 264	772.3918	1542.7690	1542.7507	11.8	0	41	0.0041	1	U	R.WINDQYVPYAFK.G
1641	253 - 264	772.3919	1542.7693	1542.7507	12.1	0	64	1.9e-005	1	U	R.WINDQYVPYAFK.G
1642	253 - 264	772.3924	1542.7703	1542.7507	12.7	0	14	1.9	1	U	R.WINDQYVPYAFK.G
1643	253 - 264	772.3927	1542.7709	1542.7507	13.1	0	29	0.061	1	U	R.WINDQYVPYAFK.G
1644	253 - 264	772.3935	1542.7724	1542.7507	14.0	0	31	0.038	1	U	R.WINDQYVPYAFK.G
1836	265 - 280	612.3009	1833.8808	1833.8574	12.8	1	17	0.89	1	U	K.GKEWVGYDDAISFGYK.A
1837	265 - 280	612.3013	1833.8820	1833.8574	13.4	1	18	0.6	1	U	K.GKEWVGYDDAISFGYK.A
1838	265 - 280	612.3015	1833.8825	1833.8574	13.7	1	36	0.011	1	U	K.GKEWVGYDDAISFGYK.A
1718	267 - 280	825.3877	1648.7609	1648.7409	12.1	0	46	0.0012	1	U	K.EWVGYDDAISFGYK.A
1719	267 - 280	825.3881	1648.7616	1648.7409	12.5	0	62	2.8e-005	1	U	K.EWVGYDDAISFGYK.A
1720	267 - 280	825.3882	1648.7619	1648.7409	12.7	0	66	1.1e-005	1	U	K.EWVGYDDAISFGYK.A
1721	267 - 280	825.3884	1648.7623	1648.7409	12.9	0	76	1.1e-006	1	U	K.EWVGYDDAISFGYK.A
1722	267 - 280	825.3894	1648.7643	1648.7409	14.2	0	13	2.1	1	U	K.EWVGYDDAISFGYK.A
405	281 - 285	625.3793	624.3721	624.3635	13.7	0	26	0.038	1		K.AFFIK.R
406	281 - 285	625.3798	624.3726	624.3635	14.5	0	18	0.29	1		K.AFFIK.R
1884	287 - 303	675.6521	2023.9345	2023.9098	12.2	0	12	3.6	1	U	R.EHFGGAMVWTLDLDDFR.G + Oxidation (M)
1885	287 - 303	675.6524	2023.9353	2023.9098	12.6	0	44	0.0022	1	U	R.EHFGGAMVWTLDLDDFR.G + Oxidation (M)
1886	287 - 303	675.6545	2023.9416	2023.9098	15.7	0	31	0.044	1	U	R.EHFGGAMVWTLDLDDFR.G + Oxidation (M)
1159	346 - 355	566.7848	1131.5550	1131.5416	11.8	0	16	1.4	1	U	R.IGPEMPTMTR.D
1160	346 - 355	566.7853	1131.5559	1131.5416	12.6	0	22	0.37	1	U	R.IGPEMPTMTR.D
1161	346 - 355	566.7853	1131.5560	1131.5416	12.7	0	33	0.033	1	U	R.IGPEMPTMTR.D
1162	346 - 355	566.7853	1131.5561	1131.5416	12.8	0	14	2.4	1	U	R.IGPEMPTMTR.D
1163	346 - 355	566.7853	1131.5561	1131.5416	12.8	0	31	0.053	1	U	R.IGPEMPTMTR.D
1164	346 - 355	566.7854	1131.5563	1131.5416	12.9	0	26	0.14	1	U	R.IGPEMPTMTR.D
1165	346 - 355	566.7854	1131.5563	1131.5416	13.0	0	2	34	1	U	R.IGPEMPTMTR.D
1166	346 - 355	566.7855	1131.5564	1131.5416	13.0	0	24	0.22	1	U	R.IGPEMPTMTR.D
1167	346 - 355	566.7855	1131.5565	1131.5416	13.1	0	34	0.027	1	U	R.IGPEMPTMTR.D
1168	346 - 355	566.7856	1131.5566	1131.5416	13.2	0	25	0.19	1	U	R.IGPEMPTMTR.D
1169	346 - 355	566.7856	1131.5566	1131.5416	13.2	0	30	0.062	1	U	R.IGPEMPTMTR.D
1170	346 - 355	566.7856	1131.5566	1131.5416	13.2	0	4	26	1	U	R.IGPEMPTMTR.D
1171	346 - 355	566.7856	1131.5566	1131.5416	13.2	0	17	1.2	1	U	R.IGPEMPTMTR.D
1172	346 - 355	566.7856	1131.5567	1131.5416	13.3	0	48	0.00088	1	U	R.IGPEMPTMTR.D
1173	346 - 355	566.7857	1131.5567	1131.5416	13.4	0	39	0.0081	1	U	R.IGPEMPTMTR.D
1174	346 - 355	566.7858	1131.5570	1131.5416	13.6	0	16	1.7	1	U	R.IGPEMPTMTR.D
1175	346 - 355	566.7858	1131.5570	1131.5416	13.6	0	29	0.08	1	U	R.IGPEMPTMTR.D
1176	346 - 355	566.7858	1131.5571	1131.5416	13.7	0	40	0.0067	1	U	R.IGPEMPTMTR.D
1177	346 - 355	566.7859	1131.5572	1131.5416	13.7	0	12	4.2	1	U	R.IGPEMPTMTR.D
1178	346 - 355	566.7859	1131.5572	1131.5416	13.7	0	4	27	1	U	R.IGPEMPTMTR.D
1179	346 - 355	566.7859	1131.5572	1131.5416	13.7	0	21	0.5	1	U	R.IGPEMPTMTR.D
1180	346 - 355	566.7859	1131.5572	1131.5416	13.8	0	29	0.078	1	U	R.IGPEMPTMTR.D
1181	346 - 355	566.7859	1131.5572	1131.5416	13.8	0	29	0.074	1	U	R.IGPEMPTMTR.D
1182	346 - 355	566.7859	1131.5573	1131.5416	13.8	0	24	0.22	1	U	R.IGPEMPTMTR.D
1183	346 - 355	566.7859	1131.5573	1131.5416	13.9	0	28	0.1	1	U	R.IGPEMPTMTR.D
1184	346 - 355	566.7860	1131.5574	1131.5416	13.9	0	42	0.0038	1	U	R.IGPEMPTMTR.D
1185	346 - 355	566.7860	1131.5574	1131.5416	14.0	0	14	2.6	1	U	R.IGPEMPTMTR.D
1186	346 - 355	566.7860	1131.5574	1131.5416	14.0	0	14	2.2	1	U	R.IGPEMPTMTR.D
1188	346 - 355	566.7861	1131.5575	1131.5416	14.1	0	20	0.61	1	U	R.IGPEMPTMTR.D
1189	346 - 355	566.7861	1131.5576	1131.5416	14.1	0	33	0.031	1	U	R.IGPEMPTMTR.D
1190	346 - 355	566.7861	1131.5576	1131.5416	14.1	0	39	0.0085	1	U	R.IGPEMPTMTR.D
1191	346 - 355	566.7861	1131.5577	1131.5416	14.2	0	12	3.5	1	U	R.IGPEMPTMTR.D
1192	346 - 355	566.7861	1131.5577	1131.5416	14.2	0	8	9.2	1	U	R.IGPEMPTMTR.D
1193	346 - 355	566.7862	1131.5579	1131.5416	14.3	0	44	0.0023	1	U	R.IGPEMPTMTR.D
1194	346 - 355	566.7862	1131.5579	1131.5416	14.4	0	23	0.29	1	U	R.IGPEMPTMTR.D
1195	346 - 355	566.7862	1131.5579	1131.5416	14.4	0	29	0.078	1	U	R.IGPEMPTMTR.D

Query	Start – End	Observed	Mr(expt)	Mr(calc)	ppm	M	Score	Expect	Rank	U	Peptide
1196	346 - 355	566.7863	1131.5579	1131.5416	14.4	0	20	0.59	1	U	R.IGPEMPTMTR.D
1197	346 - 355	566.7863	1131.5580	1131.5416	14.4	0	8	8.8	1	U	R.IGPEMPTMTR.D
1198	346 - 355	566.7863	1131.5580	1131.5416	14.5	0	36	0.015	1	U	R.IGPEMPTMTR.D
1199	346 - 355	566.7864	1131.5582	1131.5416	14.6	0	16	1.7	1	U	R.IGPEMPTMTR.D
1200	346 - 355	566.7864	1131.5582	1131.5416	14.6	0	21	0.49	1	U	R.IGPEMPTMTR.D
1201	346 - 355	566.7864	1131.5582	1131.5416	14.7	0	29	0.083	1	U	R.IGPEMPTMTR.D
1202	346 - 355	566.7864	1131.5583	1131.5416	14.7	0	17	1.2	1	U	R.IGPEMPTMTR.D
1203	346 - 355	566.7864	1131.5583	1131.5416	14.7	0	11	4.8	1	U	R.IGPEMPTMTR.D
1204	346 - 355	566.7865	1131.5584	1131.5416	14.9	0	12	4.2	1	U	R.IGPEMPTMTR.D
1205	346 - 355	566.7865	1131.5584	1131.5416	14.9	0	19	0.88	1	U	R.IGPEMPTMTR.D
1206	346 - 355	566.7865	1131.5585	1131.5416	14.9	0	32	0.044	1	U	R.IGPEMPTMTR.D
1208	346 - 355	566.7866	1131.5586	1131.5416	15.0	0	19	0.88	1	U	R.IGPEMPTMTR.D
1209	346 - 355	566.7866	1131.5586	1131.5416	15.0	0	19	0.85	1	U	R.IGPEMPTMTR.D
1210	346 - 355	566.7866	1131.5586	1131.5416	15.0	0	7	13	1	U	R.IGPEMPTMTR.D
1211	346 - 355	566.7866	1131.5586	1131.5416	15.0	0	40	0.0059	1	U	R.IGPEMPTMTR.D
1212	346 - 355	566.7866	1131.5587	1131.5416	15.1	0	3	31	1	U	R.IGPEMPTMTR.D
1213	346 - 355	566.7866	1131.5587	1131.5416	15.1	0	15	1.9	1	U	R.IGPEMPTMTR.D
1214	346 - 355	566.7867	1131.5587	1131.5416	15.1	0	6	16	1	U	R.IGPEMPTMTR.D
1215	346 - 355	566.7867	1131.5588	1131.5416	15.2	0	9	7.6	1	U	R.IGPEMPTMTR.D
1216	346 - 355	566.7867	1131.5589	1131.5416	15.2	0	17	1.2	1	U	R.IGPEMPTMTR.D
1217	346 - 355	566.7867	1131.5589	1131.5416	15.3	0	14	2.3	1	U	R.IGPEMPTMTR.D
1218	346 - 355	566.7868	1131.5590	1131.5416	15.3	0	1	51	2	U	R.IGPEMPTMTR.D
1220	346 - 355	566.7868	1131.5590	1131.5416	15.4	0	14	2.7	1	U	R.IGPEMPTMTR.D
1221	346 - 355	566.7868	1131.5591	1131.5416	15.4	0	14	2.4	1	U	R.IGPEMPTMTR.D
1222	346 - 355	566.7868	1131.5591	1131.5416	15.4	0	2	38	1	U	R.IGPEMPTMTR.D
1223	346 - 355	566.7868	1131.5591	1131.5416	15.4	0	4	26	1	U	R.IGPEMPTMTR.D
1224	346 - 355	566.7868	1131.5591	1131.5416	15.4	0	24	0.27	1	U	R.IGPEMPTMTR.D
1225	346 - 355	566.7869	1131.5592	1131.5416	15.5	0	30	0.065	1	U	R.IGPEMPTMTR.D
1226	346 - 355	566.7869	1131.5593	1131.5416	15.6	0	3	35	1	U	R.IGPEMPTMTR.D
1227	346 - 355	566.7869	1131.5593	1131.5416	15.6	0	19	0.74	1	U	R.IGPEMPTMTR.D
1228	346 - 355	566.7870	1131.5594	1131.5416	15.7	0	15	1.9	1	U	R.IGPEMPTMTR.D
1229	346 - 355	566.7871	1131.5596	1131.5416	15.9	0	13	3.3	1	U	R.IGPEMPTMTR.D
1231	346 - 355	566.7871	1131.5597	1131.5416	16.0	0	19	0.74	1	U	R.IGPEMPTMTR.D
1232	346 - 355	566.7872	1131.5598	1131.5416	16.0	0	34	0.026	1	U	R.IGPEMPTMTR.D
1233	346 - 355	566.7872	1131.5598	1131.5416	16.1	0	10	7	1	U	R.IGPEMPTMTR.D
1234	346 - 355	566.7872	1131.5598	1131.5416	16.1	0	16	1.5	1	U	R.IGPEMPTMTR.D
1235	346 - 355	566.7873	1131.5600	1131.5416	16.2	0	12	3.7	1	U	R.IGPEMPTMTR.D
1261	346 - 355	574.7828	1147.5510	1147.5366	12.6	0	37	0.0098	1	U	R.IGPEMPTMTR.D + Oxidation (M)
1262	346 - 355	574.7832	1147.5519	1147.5366	13.4	0	8	7.7	1	U	R.IGPEMPTMTR.D + Oxidation (M)
1263	346 - 355	574.7837	1147.5528	1147.5366	14.2	0	29	0.069	1	U	R.IGPEMPTMTR.D + Oxidation (M)
1264	346 - 355	574.7839	1147.5533	1147.5366	14.6	0	36	0.014	1	U	R.IGPEMPTMTR.D + Oxidation (M)
1265	346 - 355	574.7845	1147.5545	1147.5366	15.7	0	15	1.8	1	U	R.IGPEMPTMTR.D + Oxidation (M)
1291	346 - 355	582.7811	1163.5476	1163.5315	13.8	0	17	1	1	U	R.IGPEMPTMTR.D + 2 Oxidation (M)
1292	346 - 355	582.7815	1163.5484	1163.5315	14.6	0	6	12	1	U	R.IGPEMPTMTR.D + 2 Oxidation (M)

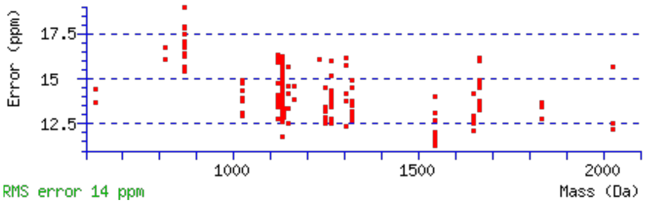

Mascot: http://www.matrixscience.com/
